# Usefulness of 3D-surgical guides in breast conserving surgery after neoadjuvant treatment

**DOI:** 10.1038/s41598-021-83114-2

**Published:** 2021-02-09

**Authors:** Han Shin Lee, Hee Jeong Kim, Il Yong Chung, Jisun Kim, Sae Byul Lee, Jong Won Lee, Byung Ho Son, Sei Hyun Ahn, Hak Hee Kim, Joon Beom Seo, Jin Hee Ahn, Gyungyub Gong, Sangwook Lee, Namkug Kim, Beom Seok Ko

**Affiliations:** 1 Department of Surgery, Gyeongsang National University School of Medicine and Gyeongsang National University Changwon Hospital, Changwon, Republic of Korea; 2 Division of Breast Surgery, Department of Surgery, University of Ulsan College of Medicine, Asan Medical Center, Seoul, Republic of Korea; 3 Department of Radiology, University of Ulsan College of Medicine, Asan Medical Center, Seoul, Republic of Korea; 4 Department of Oncology, University of Ulsan College of Medicine, Asan Medical Center, Seoul, Republic of Korea; 5 Department of Pathology, University of Ulsan College of Medicine, Asan Medical Center, Seoul, Republic of Korea; 6 Department of Convergence Medicine, University of Ulsan College of Medicine, Asan Medical Center, Seoul, Korea

**Keywords:** Medical research, Oncology

## Abstract

We used 3D printed-breast surgical guides (3DP-BSG) to designate the original tumor area from the pre-treatment magnetic resonance imaging (MRI) during breast-conserving surgery (BCS) in breast cancer patients who received neoadjuvant systemic therapy (NST). Targeting the original tumor area in such patients using conventional localization techniques is difficult. For precise BCS, a method that marks the tumor area found on MRI directly to the breast is needed. In this prospective study, patients were enrolled for BCS after receiving NST. Partial resection was performed using a prone/supine MRI-based 3DP-BSG. Frozen biopsies were analyzed to confirm clear tumor margins. The tumor characteristics, pathologic results, resection margins, and the distance between the tumor and margin were analyzed. Thirty-nine patients were enrolled with 3DP-BSG for BCS. The median nearest distance between the tumor and the resection margin was 3.9 cm (range 1.2–7.8 cm). Frozen sections showed positive margins in 4/39 (10.3%) patients. Three had invasive cancers, and one had carcinoma in situ; all underwent additional resection. Final pathology revealed clear margins. After 3-year surveillance, 3/39 patients had recurrent breast cancer. With 3DP-BSG for BCS in breast cancer patients receiving NST, the original tumor area can be identified and marked directly on the breast, which is useful for surgery. *Trial Registration*: Clinical Research Information Service (CRIS) Identifier Number: KCT0002272. First registration number and date: No. 1 (27/04/2016).

## Introduction

Breast cancer is the most common malignant tumor found in women worldwide^1^. With the development of diagnostic breast imaging and the popularization of screening programs, the diagnosis of early breast cancer and the occurrence of breast-conserving surgery (BCS) have increased^2^. In early breast cancer, BCS has been established as a standard procedure due to the lack of prognostic difference between radiation therapy after partial excision and mastectomy^3^. As the breast is a cosmetically important organ in women, BCS should be attempted first, if possible. In cases where the tumor size is already large at the time of the discovery, a mastectomy may be necessary. In breast cancer, neoadjuvant systemic therapy (NST) is known to have a similar prognosis as adjuvant treatment^4^. However, in cases where the tumor size is too large for BCS at diagnosis, NST has been performed prior to attempting BCS^5^. Surgeons expected improved cosmetic results when performing BCS after NST due to decreased tissue removal, but a review disqualified this belief^6^. Positive margins at BCS are closely related to local recurrence, so adequate margins and total tumor excision are vital^7^. Precise tumor removal requires techniques that accurately identify and indicate the tumor area. Imaging techniques such as mammography (MMG), ultrasonography (USG), and magnetic resonance imaging (MRI) are commonly used to obtain information about tumors. Among these, MRI is known to be the most accurate in detecting the extent of residual cancer after NST^8,9^. Several localization methods have been used to remove tumors that are challenging to identify with breast palpation alone. The most commonly used method, the MMG- or USG-guided wire localization (WL), may be associated with complications such as vasovagal syncope and pneumothorax, leading to problems during surgery like migration, cutting, and loss^10^. In addition, this procedure makes quantification of the tumor difficult^10,11^. New localization methods such as radio-guided occult lesion localization (ROLL) and radioactive seed localization (RSL) have been developed due to the limitations of WL. These methods have been useful in the partial tumor resection in patients who underwent NST^12^. Several breast imaging and localization techniques have been used to perform BCS after NST, but local recurrence rates have been higher than those in patients who underwent surgery first^13^. This is due to the difficulty in identifying the original tumor area from pre-treatment MRIs, and in marking the area directly on the breast. To solve this problem, a supine MRI-based 3D printed-breast surgical guide (3DP-BSG) was developed. We report the findings from using 3DP-BSG for patients receiving BCS after NST.

## Methods

### Patient eligibility

This trial was designed as a prospective single-institution cohort study. Institutional review board approval and informed consent from all enrolled patients were obtained. Our study protocol was approved by Institutional Review Board of Asan Medical Center, Seoul, Korea (IRB No. 2016-0326). The trial registration and Clinical Research Information Service (CRIS) identifier number is KCT0002272. And first registration number and date is No. 1 (27/04/2016). Women between the ages of 20 and 65 with pathologically confirmed invasive cancer and expected to undergo BCS after NST, were enrolled in the study. Patients with multiple tumors or large tumors who were expected to undergo mastectomy even after NST, were excluded. Patients with claustrophobia, adverse reactions to gadolinium, or with contraindications for MRI were also excluded. Patients participating in the study underwent a supine MRI series along with a baseline breast MRI. In total, 47 patients were enrolled in the study, and the final 39 patients underwent BCS with 3DP-BSG.

### MRI acquisition and production of the surgical guide

**Figure 1:**
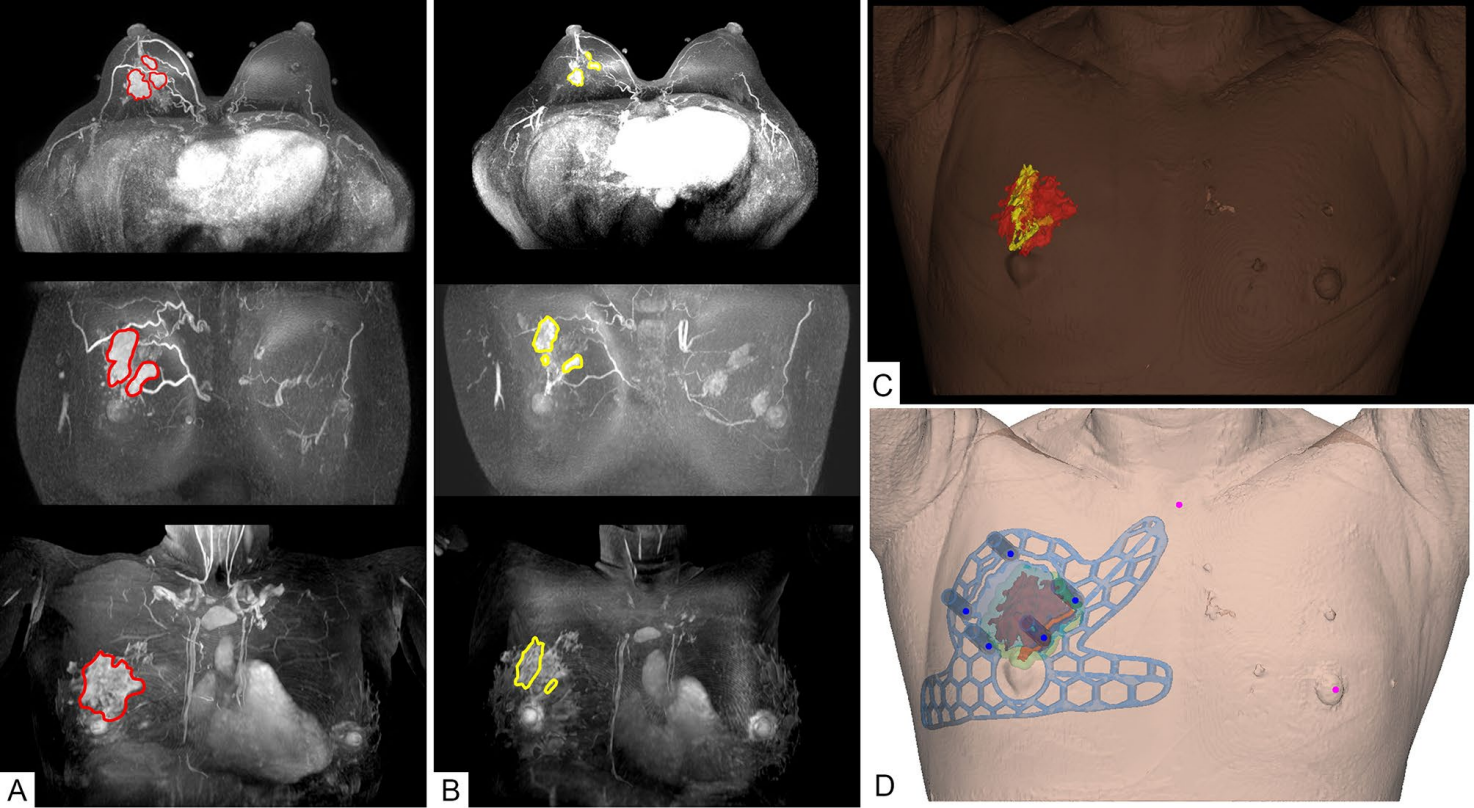
Three-dimensional modeling of the breast and tumor area based on magnetic resonance imaging (MRI) in neoadjuvant systemic therapy (NST) patient and patient-specific tumor target 3D printed-breast surgical guide (3DP-BSG) modeling. (**A**) 3D modeling of the breast and tumor area using MRI before NST. (**B**) 3D modeling of the breast and tumor area using MRI after NST. (**C**) 3D modeling of the breast and tumor area using MRI fusion of the images obtained before and after NST. D. Patient-specific 3D-BSG modeling using three-dimensional information about breasts and tumors.

A bilateral breast MRI was performed before and after NST using a 1.5-T MRI system (Magnetom Avanto, Siemens Healthineers) and a dedicated breast surface coil in the prone position. A supine MRI was included to obtain breast images in the surgical position. The original tumor area and breast were three-dimensionally modeled by combining MRI data obtained before and after treatment (Fig. 1A–C). In addition, the 3D-shape and the safety excision margin were designed by combining the modeled image and the 3D-images that were constructed based on the MRI data. The prepared digital model was saved in a stereo-lithography file format before being exported to a 3D printer (Connex3 Object500; Stratasys Corporation, Rchovot, Israel) for the surgical guide creation (Fig. 1D).

**Figure 2:**
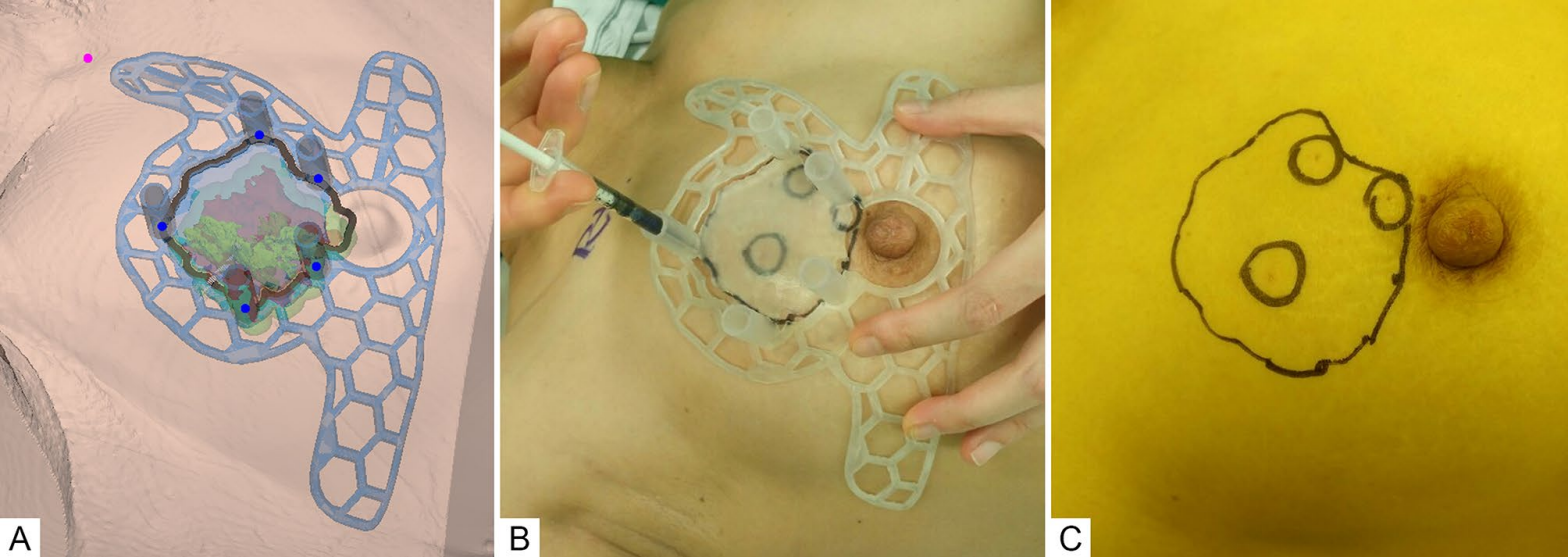
Tumor-targeting with patient-specific 3D printed-breast surgical guide (3DP-BSG) after neoadjuvant systemic therapy (NST). (**A**) MRI-based patient-specific 3DP-BSG modeling. (**B**) Marking of the tumor area on the skin surface using 3DP-BSG, and target the tumor inside the breast by injecting blue dye. (**C**) Tumors observed in USG (three inner circles) after NST and the original tumor area (external boundary) using 3DP-BSG.

The 3DP-BSG was modeled to be 0.5 cm outside the tumor edges to ensure free margins. To ensure accurate display of the tumor range, the 3DP-BSG was modeled according to the following guidelines: (1) the model fits on the skin surface of the breast, (2) includes a hole at the nipple, and (3) has direction marks indicating the opposite nipple and the suprasternal notch to prevent rotation of the 3DP-BSG. In addition to allowing for depictions of tumor range on the breast surface, the 3DP-BSG model included a column to guide the blue dye injection for an accurate indication of the tumor extent within the breast (Fig. 2).

### Operation and pathologic assessments

After general anesthesia, the arm was superiorly positioned for axillary surgery, and the prepared 3DP-BSG was applied. The 3DP-BSG was placed on the breast with the bilateral nipples and suprasternal notch utilized as landmarks. The groove of the 3DP-BSG, modeled along the boundary of the tumor, was used to draw the tumor area over the breast skin. A blue dye was injected via the 3DP-BSG column into the breast to mark the tumor borders internally (Fig. 2). The breast tissue, abnormal or otherwise, was removed following the tumor border, as indicated by the blue dye. Ties of different lengths at the 3 o'clock and 12 o'clock positions of the removed breast tissue indicated the tumor orientation. Tissue from several sites of the surgical cavity was extracted for frozen biopsy to analyze for residual cancer. In the case of any tumor-positive results, re-excision was performed. The lack of breast tissue continuity was not considered to affect recurrence and was excluded from the evaluation of the resection margin^14^. The distance from the tumor edge to the resection margin was measured at the 3, 6, 9, and 12 o'clock positions. A sentinel node biopsy (SNB) was performed depending on the cancer type, and an axillary lymph node dissection (ALND) was performed according to the presence of node metastasis.

### Ethical approval

All procedures performed in studies involving human participants were in accordance with the ethical standards of the institutional and/or national research committee and with the 1964 Helsinki declaration and its later amendments or comparable ethical standards.

## Results

Table 1 describes the patient and tumor characteristics. From July 2016 to January 2017, 39 patients were enrolled. The median age of the patients was 46 years (range 34–61 years). In 23 cases, only SNB was performed. In 16 cases, metastatic nodes were found, mandating ALND. In one case where only a small-sized ductal carcinoma in situ (DCIS) was detected, axillary surgery was not performed. After NST, eight of 39 patients (20.5%) were deemed to have pathological complete response (pCR) status. Most tumors (89.7%) were removed with adequate surgical margins, as summarized in Table 2. In four cases, frozen biopsies of the resection margins were positive, three for invasive cancer, and one for carcinoma in situ. Re-excision was performed with final pathology, revealing that all margins were clean; thus, there was no conversion to mastectomy. The median size of the long axis of the tumor was 1.2 cm (range 0–4.5 cm), and the median size of the long axis of the removed breast tissue was 6.5 cm (range 4.5–13.0 cm). The median distance between the tumor and the resection margin was 3.9 cm (range 1.2–7.8 cm). The median operation time was 80 min in the SNB group and 101.5 min in the ALND group. A 3-year follow-up revealed that three patients had breast cancer recurrence.

**Table 1:** Patient and tumor characteristics.

Total patients (N = 39)	N (%)
**Age (yrs)**	
≤ 50	26 (67.7)
> 50	13 (32.3)
**Pathology**	
Invasive ductal carcinoma	37 (94.9)
Etc	2 (5.1)
**Clinical T stage**	
T1	2 (5.1)
T2	32 (82.1)
T3	5 (12.8)
**Clinical N stage**	
N0	23 (59.0)
N1	13 (33.3)
N2	2 (5.1)
N3	1 (2.6)
**Tumor grade**	
I	0
II	28 (71.8)
III	11 (28.2)
**Multifocality (image finding)**	
Yes	17 (43.6)
No	22 (56.4)
**Multifocality (pathologic)**	
Yes	5 (12.8)
No	34 (87.2)
**Subtype**	
HR(+), HER2(−)	21 (53.8)
HR(+), HER2(+)	5 (12.9)
HR(−), HER2(+)	7 (17.9)
HR(−), HER2(−)	6 (15.4)

**Table 2:** Operative pathologic findings and surveillance.

Total patients (N = 39)	N (%)
**Margin status (frozen)**	
Negative	35 (89.7)
Positive	4 (10.3)
**Margin status (permanent)**	
Negative	39 (100.0)
Positive	0 (0.0)
**Axillary surgery**	
SLNB	23 (59.0)
ALND	16 (41.0)
**Lymph node status**	
Positive	15 (38.5)
Negative	24 (61.5)
**Mean operation time (min)**	
SNB only-Median	80
SNB only-range	52–110
ALND-median	101
ALND-range	43–172
**Nearest margin (cm)**	
Mean (SD)	4.2 (1.7)
Median	3.9
Range	1.2–7.8
**Tumor diameter (cm)**	
Mean (SD)	1.31 (1.2)
Median	1.2
Range	0–4.5
**Specimen diameter (cm)**	
Mean (SD)	6.9 (1.9)
Median	6.5
Range	4.5–13.0
**Difference of size**	
Mean (SD)	− 2.0 (1.7)
Median	− 1.8
Range	− 6.2 to 0.7
**3 year follow-up**	
Recurrence	3 (7.7)

In contrast to the MMG- or USG-guided targeting methods, the 3DP-BSG can quantitatively indicate the extent of the tumor in the breast on MRI. The advantage of using this method is that an area of the breast from a past MRI can be summoned, and this area can be marked on the present breast. In the 3DP-BSG method, a guide which is made based on MRI is placed on the breast after the target patient is under general anesthesia, and the outline of the tumor is drawn on the skin according to the groove marked on the guide and the column of the guide, to target the tumor inside the breast. A blue dye is injected after passing through the syringe.

## Discussion

Since breasts are cosmetically important to women, partial excision is attempted in patients with breast cancer, if possible. It is widely known that sexual and psychosocial outcomes are better in patients who undergo BCS than in those who undergo mastectomy^15^. When performing BCS, securing adequate margins for complete tumor removal while maximizing cosmetic results is necessary. These outcomes can be achieved by ensuring minimal breast deformity and asymmetry by preserving the maximal volume of normal breast tissue. NST can convert inoperable tumors into operable ones, provide early information on tumor response to treatment, and reduce the administration of chemotherapy in the absence of treatment response^16^. In large tumors, NST is performed for size reduction in an attempt to preserve the breast. According to previous literature, however, the occurrence rate of tumor-positive resection margins in BCS after NST is 40%^6^. According to a report by the Early Breast Cancer Trialists' Collaborative Group (EBCTCG), the local recurrence rate was higher in patients who received BCS after NST than in those who received adjuvant therapy after BCS^13^. Volders et al*.* reviewed 26 papers on patients who received BCS after NST and reported that they could not demonstrate the benefit of NST in terms of removed breast volume or cosmetic results^6^. To improve these outcomes, the tumor shape must be accurately identified for precise tumor removal. MRI is the most accurate way to determine the tumor extent in breast cancer patients, and can even accurately identify areas of residual tumor after NST^8,17^. It is difficult to mark the extent of the tumor noted on the MRI directly onto the breast; however, several methods have been attempted to achieve this end. Yamashiro et al*.* reported a useful MRI marking technique from supine MRI in 14 patients who underwent BCS^18^. Sakakibara et al*.* described the success of BCS in patients with ductal carcinoma in situ using a projection technique based on supine MRI information^19^. Pallone et al*.* suggested using supine MRI and 3D optical scans to improve surgical guidance by matching the breast surface and segmented breast information^20^. None of these methods, however, can display the area of the original tumor. The numerous attempts to mark the range of tumors set to be removed in patients with NST are documented in Table 3. Rubio et al*.* reported a negative resection margin rate of 86.6% in 45 breast cancer patients by using WL at BCS after NST^21^. In addition, Volders et al*.* reported a tumor-free margin of 72.7% in 626 breast cancer patients who underwent WL at BCS after NST^22^. A tattoo method for localizing the non-palpable breast lesions after NST is also available. Mathieu et al*.* demonstrated that a charcoal suspension injection easily visualized the tumor specimen when 10 of 91 patients were free of macroscopic residual tumor^23^. The simplest method is to insert a radiopaque clip into the tumor margin before treatment. However, the disadvantages with this technique include placement difficulty, migration, loss, failure, and patient pain. Success also depends on the type of tissue marker clips used, since metallic clips can cause artifacts and reduce MRI sensitivity^24^. Oh et al*.* reported a negative resection margin rate of 89% using radiopaque clips in breast cancer patients who were receiving NST and BCS^25^. Gobardhan et al*.* reported complete resection in 92% of unifocal and multifocal breast tumors in 85 cases of breast cancer using RSL at BCS after NST^26^. Donker et al*.*, however, reported a pathologically positive margin rate of 13% in 71 breast cancer patients using RSL at BCS after NST^12^. The RSL method, using a gamma detector, allows for easier localization than does metallic clip localization. However, this method also has disadvantages such as migration, loss, and pain, along with difficulty in management, and involvement of high rates of radiation exposure^27^. Ramos et al*.* reported unclear margins in 12% of 58 breast cancer patients who underwent intraoperative ultrasound excision (IOUS) after NST^28^. Furthermore, Rubio et al*.* reported negative resection margins in 87.5% of 112 breast cancer patients using the IOUS lumpectomy method after NST^21^. Recently, a method involving the pre-treatment insertion of multiple clips to mark the area of the original tumor that is difficult to define after NST has been implemented; however, this did not improve results. To overcome this issue, Wu et al. proposed the use of 3DP-BSG that can quantitatively indicate the extent of tumors on MRI^29^. Conventional tumor targeting methods employed so far have limitations that cannot be overcome, as follows: (1) these methods cannot quantitatively mark the tumor area on the breast on MRI; and (2) cannot summon the original tumor area. However, 3DP-BSG solves this problem and enables accurate localization compared to the other targeting methods. Theoretically, when using 3DP-BSG, the tumor should be completely and precisely removed. In our study, 10.3% of the patients had positive margins. This was likely due to the tumor actual size being larger than that observed on MRI, due to MRI limitations. Additionally, our study showed a 7.7% recurrence rate after a 3-year follow-up. A meta-analysis of ten randomized trials performed with BCS after NST^13^ reported a 5-year local recurrence rate of 12.1%. Although the follow-up period was short in our study, the obtained results were promising. Compared to other conventional localization techniques, 3DP-BSG has many advantages. First, the tumor area noted on the MRI can be marked both within the breast and on the skin. Second, in patients receiving NST, the area of the original tumor before treatment can be marked. Third, BSG is a localization technique that does not cause pain in the patient. Fourth, since the 3DP-BSG is made prior to surgery, it does not require changes in the surgery schedule or increased preparation time for localization. Fifth, there is no risk of radiation exposure. Sixth, there is no risk of migration, loss, or cutting. In addition, the use of a 3DP-BSG allows for the preservation of normal breast tissue and precise tumor removal, enhancing the cosmetic effect. We expect that even beginners can easily overcome the BCS learning curve.

**Table 3:** Localization techniques in patients who underwent neoadjuvant systemic therapy (NST).

Localization technique	Clear margin rate	Original extension	Using MRI (quantitative)	No pain	NO procedure time	No radiation
BSG	89.7–100% ^this article^	O	O	O	O	O
WL	72.7–86.6%^21,22^	X	X	X	X	△
Carbon	81.0–89%^10,23^	X	X	X	X	△
Clip	89.0%^25^	△	X	X	X	△
RSL	87–92.0%^12,26^	△	X	X	X	X
IOUS	87.5–88%^21,28^	O	X	O	X	O
Cavity shave	91–94%^10^	X	X	O	X	O

*BSG* breast surgical guide, *WL* wire localization, *Carbon* carbon marking, *Clip* clip marker localization, *RSL* radioactive seed localization, *IOUS* intraoperative ultrasound excision, *Time* procedure time.

In conclusion, utilizing 3DP-BSGs in patients undergoing BCS after NST revealed a low rate of tumor-positive margins. By using 3DP-BSG as a localization method in patients receiving NST, it is possible to precisely target the original tumor area observed in the pre-treatment MRI. In addition, the advantages of the method are that it is painless, does not include radiation, and does not increase the procedure time.
